# Physical and thermodynamic characterization of the rice gibberellin receptor/gibberellin/DELLA protein complex

**DOI:** 10.1038/s41598-018-35765-x

**Published:** 2018-12-07

**Authors:** Hongyu Xiang, Hideyasu Okamura, Yuichiro Kezuka, Etsuko Katoh

**Affiliations:** 1 Advanced Analysis Center, National Agriculture and Food Research Organization, Tsukuba, Ibaraki, 305-8517 Japan; 2 School of Pharmacy, Iwate Medical University, Yahaba, Iwate, 028-3694 Japan; 3 Present Address: School of Life Sciences, Jilin University, Qianjin Street, Changchun, 130012 China; 4 Present Address: RIKEN Center for Biosystem Dynamics Research Laboratory for Cellular Structural Biology, Tsurumi-ku, Kanagawa 230-0045 Japan

## Abstract

Gibberellins (GAs) are phytohormones that regulate various developmental processes in plants. The initial GA signalling events involve the binding of a GA to the soluble GA receptor protein GID1, followed by the binding of the complex to the negative transcriptional regulator of GA signaling, the DELLA protein. Although X-ray structures for certain *Arabidopsis* GID1/GA/DELLA protein complexes have previously been determined, examination of these complexes did not fully clarify how a DELLA protein recognizes and binds to a GID1/GA complex. Herein, we present a study aimed at physically defining, via a combination of gel chromatography, isothermal titration calorimetry (ITC), small-angle X-ray scattering experiments (SAXS), NMR spectroscopy and mutagenesis, how the rice DELLA protein (SLR1) binds to the rice GID1/GA complex. We have identified the shortest SLR1 sequence (M28-A112) that binds the rice GID/GA complex tightly. The binding constant for the ternary complex that includes SLR1(M28-A112) is 2.9 × 10^7^ M^−1^; the binding is enthalpically driven and does not depend on the chemical nature of the bound GA. Furthermore, the results of SAXS, ITC, and gel filtration experiments indicate that when free in solution, SLR1(M28-A112) is a natively unfolded protein. The NMR experiments expand this observation to show that the unfolded mutant also contains a small amount of marginally stable secondary structure. Conversely, the protein has a highly ordered structure when bound one-to-one to GID1/GA.

## Introduction

The gibberellins (GAs) are a large family of tetracyclic diterpenoid plant hormones that induce a wide range of plant growth responses, including seed germination, stem elongation, leaf expansion, induction of flowering, and pollen maturation^1,2^. To date, over 100 different GAs have been isolated from plants, fungi, and bacteria. Although many of the GA biosynthetic and catabolic pathways are well characterized^3^, much less is known about the plant growth pathways that are initiated by GA signalling.

During the past decade, a number of rice proteins involved in GA signalling pathways have been identified by screening for rice (*Oryza sativa*) variants with aberrant growth characteristics owing to genetic mutations (reviewed in^4–6^). Specifically, the GA receptor protein (OsGID1)^7^, the DELLA protein (SLR1)^8^, and the F-box protein (GID2)^9,10^ have been shown to be essential for GA signalling in rice. Physiological and biochemical analyses of these proteins have enabled us to construct a model for the GA signalling pathway^4,11^. Accordingly, a GA first binds OsGID1. The OsGID1/GA complex then binds SLR1, a negative transcriptional regulator of GA. The interaction of the OsGID1/GA complex with SLR1 results in the degradation of SLR1 mediated by the GID2 in the SCF^GID2^ complex. Because SLR1 inhibits responses to GA signalling, SLR1 must be removed by proteolysis—which occurs when SLR1 is bound to an OsGID1/GA complex—before the GA signal can be transmitted farther down the signalling pathway.

The SLR1 sequence contains the N-terminal DELLA (residues 38–43) and TVHYNP (residues 91–96) motifs and a C-terminal GRAS domain, whose presence suggests that SLR1 is a GRAS-type transcriptional factor^12^. To date, most of the current knowledge about the structural interactions between a GA receptor and a DELLA protein is derived from two crystal structures of the corresponding *Arabidopsis* proteins AtGID1a and the truncated DELLA protein variant GAI(Q11-Q113)^13^. Consequently, much remains to be learned about the molecular recognition mechanisms that occur during the initial steps of GA-mediated plant growth pathways. The rice proteins OsGID1 and SLR1 are suitable models for such studies because they can be isolated as recombinant proteins in quantities large enough for *in vitro* structural studies.

While the three-dimensional structure of a protein complex provides detailed molecular information concerning the intermolecular interactions that are important for maintaining a complex, other methods provide complementary and supplementary information. Herein, using a combination of gel chromatography, isothermal titration calorimetry (ITC), small-angle X-ray scattering (SAXS) measurements, and NMR spectroscopy, we report an examination of various solution OsGID1/GA/SLR1 complexes that differ according to the SLR1 variant present and the isolated components in solution. We identify the shortest SLR1 sequence (M28-A112) that binds an OsGID1/GA_3_ complex with high affinity (*K*_b_ = 2.9 × 10^7^ M^−1^) and showed that their binding is enthalpically driven. Additionally, we find that the binding is independent of all tested bioactive GAs. Finally, NMR, SAXS, and gel filtration experiments show that SLR1(M28-A112) is natively unfolded, contains some secondary structure, and dimerizes when free in solution. On the other hand, SLR1(M28-A112) forms a highly ordered structure when bound one-to-one to OsGID1/GA_3_.

## Results

### Gel filtration co-chromatographies of Trx-OsGID1/GAs and Trx-SLR1 variants

**Figure 1:**
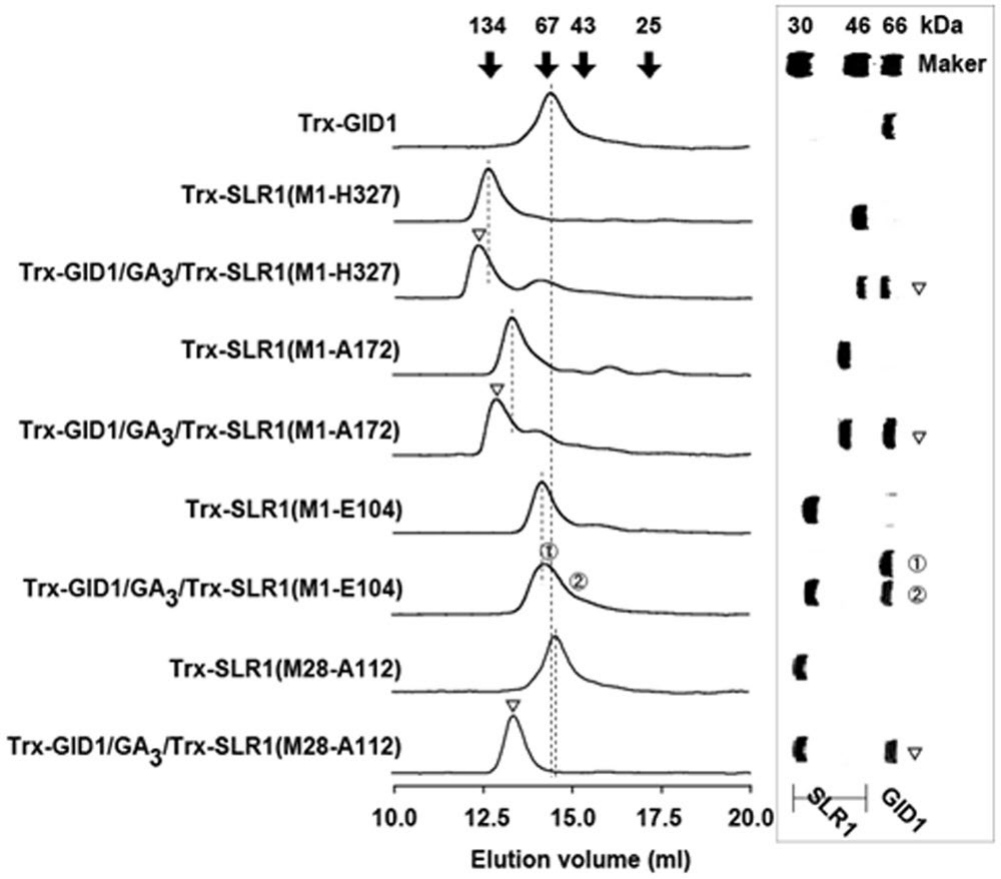
Superdex 200 gel filtration chromatograms of Trx-OsGID1/GA_3_ and various Trx-SLR1 mutants in isolation and together. The eluent was 20 mM sodium phosphate (pH 7.5), 150 mM NaCl, 2 mM β-ME, and 0.1 mM GA_3_. The protein(s) contained in the fractions indicated by the inverted open arrows and by the circled numbers were subjected to SDS-PAGE and identified by their electrophoretic migration patterns (right panel). The positions of the molecular weight standards are shown at the top of both panels.

We previously monitored the *in vitro* interactions between the Trx-OsGID1/GA_3_ complex and truncated versions of Trx-SLR1 by gel filtration^11^. As we previously showed that the Trx-tag did not affect complex formation (Fig. S1), the two newly constructed truncated versions of Trx-SLR1—used for this report to characterize the shortest possible SLR1 sequence that binds the Trx-OsGID1/GA_3_ complex—also retained the Trx-tag. These Trx-SLR1 mutants are Trx-SLR1(M1-L110) and Trx-SLR1(M28-A112). Figure 1 shows the gel filtration chromatograms for mixtures of various combinations of a Trx-GID1/GA_3_ complex and a truncated Trx-SLR1 mutant, as well as the chromatograms of the proteins in isolation. As reported previously^11^, all of the truncated SLR1 mutants elute as proteins of much larger size than would be predicted on the basis of their estimated molecular weights (MWs). It appears from the chromatograms that, with the exception of Trx-SLR1(M1-E104), all of the Trx-SLR1 mutants bind Trx-OsGID1/GA_3_; however, as the results of the isothermal titration calorimetry experiment show, SLR1(M1-E104) does bind the OsGID1/GA_3_ complex, albeit with a binding constant that is only approximately 0.4% of that found for SLR1(M28-A112) (see below). The Trx-SLR1(M28-A112) mutant, which is truncated both N- and C-terminally, elutes as a larger species (ca. 60.0 kDa) than would be predicted using the MW estimated by SDS-PAGE (ca. 30 kDa) or its theoretical monomeric MW (29.3 kDa) (Fig. 1). The estimated sizes of Trx-SLR1(M28-A112) and Trx-SLR1(M1-E104) determined by gel filtration indicate that they dimerize, while the other truncated SLR1 deletion mutants examined here and in our previous report appear to oligomerize to greater extents^11^. When Trx-SLR1(M28-A112) and Trx-OsGID1/GA_3_ are co-chromatographed, peaks at the expected positions of the two proteins are not detected, while a peak (MW ca. 91.8 kDa) eluting at the position expected for the one-to-one heterocomplex (89.3 kDa) is found. This species appears to be stable during the chromatography. Therefore, SLR1(M28-A112) is the shortest SLR1 sequence identified to date that binds Trx-OsGID1/GA_3_ tightly.

**Figure 2:**
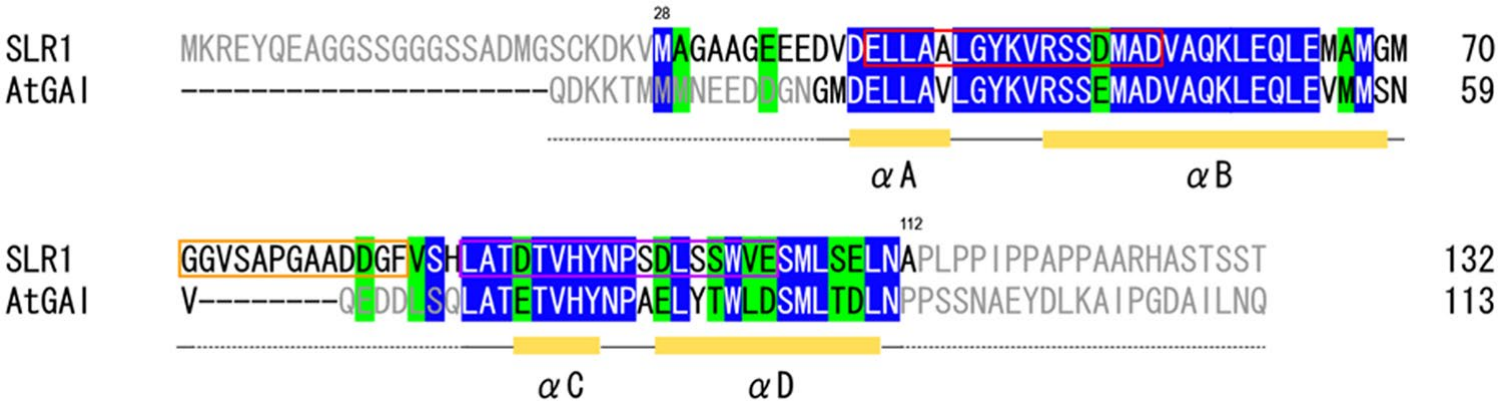
Partial sequence alignment of SLR1 and AtGAI. The aligned sequences include the shortest SLR1 sequence now known to bind OsGID1/GA_3_ tightly and the four α-helices of GAI that physically contact AtGID1a/GA^13^. The positions of the GAI α-helices are shown at the bottom. Conserved and conservatively replaced residues are highlighted in blue and green, respectively.

Murase *et al*. structurally characterized the *Arabidopsis*-derived ternary complexes AtGID1a/GA_3_/GAI(Q11-Q113) and AtGID1a/GA_4_/GAI(Q11-Q113), where GAI(Q11-Q113) is a truncated DELLA protein^13^. Examination of these complexes shows that certain residues (N25-N92 of GAI) that form four α-helices are involved in intermolecular contacts with AtGID1a (Figs 2, S2). Figure 2 shows a sequence alignment of the SLR1 M28-A112 and the GAI Q11-Q113 sequences. The residues of the four GAI helices are highly conserved in the SLR1 sequence, even though the latter sequence has an eight-residue insertion between the second and the third helices. Our characterization of the shortest SLR1 sequence that binds OsGID1 tightly, as well as its high sequence homology with the helical sequences of GAI(Q11-Q113), unequivocally establishes the SLR1 sequence that binds OsGID1.

### Small-angle X-ray scattering profiles of SLR1(M28-A112), OsGID1/GA3, and the OsGID1/GA3/SLR1(M28-A112) complex

To further characterize the overall shapes of SLR1(M28-A112), OsGID1/GA_3_, and the ternary complex, we measured the SAXS profiles of the Trx-free forms of these proteins (Fig. S3). The scattering profiles of OsGID1/GA_3_, SLR1(M28-A112), and OsGID1/GA_3_/SLR1(M28-A112) are all linear in the small *q*^2^ region of their Guinier plots (Fig. S3A). The calculated radius of gyration (*R*_g_) values for OsGID1/GA_3_ and SLR1(M28-A112) are 31.3 ± 0.1 Å and 30.4 ± 0.3 Å, respectively. The estimated molecular weight of OsGID1/GA_3_ is then 81.2 kDa, suggesting that OsGID1 dimerizes (theoretical MW of dimeric OsGID1, 79.2 kDa) in solution. The sample concentration of OsGID1/GA_3_ was 3.0 mg/ml for the SAXS measurements and approximately 0.5 mg/ml for the gel filtration chromatographies; therefore, a monomer/dimer OsGID1/GA_3_ equilibrium probably exists that favours the dimeric state as the protein concentration increases. The molecular weight of SLR1(M28-A112) is estimated to be 12.9 kDa, which is approximately 1.5-fold larger than the theoretical value (8.9 kDa). Information about molecular shape can be obtained using the entire scattering profile in a Kratky plot. The presence of a peak in a Kratky plot indicates that a protein has a globular shape, while a plateau between 0.1 and 0.3 Å^−1^ indicates that an unfolded protein exists^14^. The plots of Fig. S3B clearly indicate that OsGID1/GA_3_ is globular and that SLR1(M28-A112) is unfolded. The larger than expected *R*_g_ value for SLR1(M28-A112), given its MW, is obviously a consequence of an unfolded structure.

The Guinier plot of OsGID1/GA_3_/SLR1(M28-A112) gives an *R*_g_ value of 23.5 ± 0.1 Å, which is smaller than the 31.3 ± 0.1 Å value found for OsGID1/GA_3_. The MW of the ternary complex, estimated using *I*(0), is 40.5 kDa, which is similar to that expected for a one-to-one complex of OsGID1/GA_3_ and SLR1(M28-A112) (48.5 kDa). The presence of the peak in the Kratky plot suggests that OsGID1/GA_3_/SLR1(M28-A112) has a globular shape (Fig. S3B). (The maximum value of a peak for a globular protein in a Kratky plot should be approximately 3*I*(0)/(*eR*_g_^2^) at *q* = *R*_g_, where *e* denotes the base of the natural logarithm^15,16^). The *R*_g_ values of OsGID1/GA_3_ and OsGID1/GA_3_/SLR1(M28-A112) calculated using the data from the Kratky plots are 32.1 Å and 24.6 Å, which are in agreement with the values obtained from the Guinier plots (31.3 ± 0.1 Å and 23.5 ± 0.1 Å). Taken together, the *R*_g_ values, calculated molecular weights, and Kratky plots strongly suggest the following: OsGID1/GA_3_ and SLR1(M28-A112) form a 1:1 complex with a globular shape; as an isolated species, OsGID1/GA_3_ has a propensity to dimerize; and SLR1(M28-A112) is a natively unfolded protein.

### Thermodynamics of OsGID1/GA and SLR1 binding monitored by ITC

**Figure 3:**
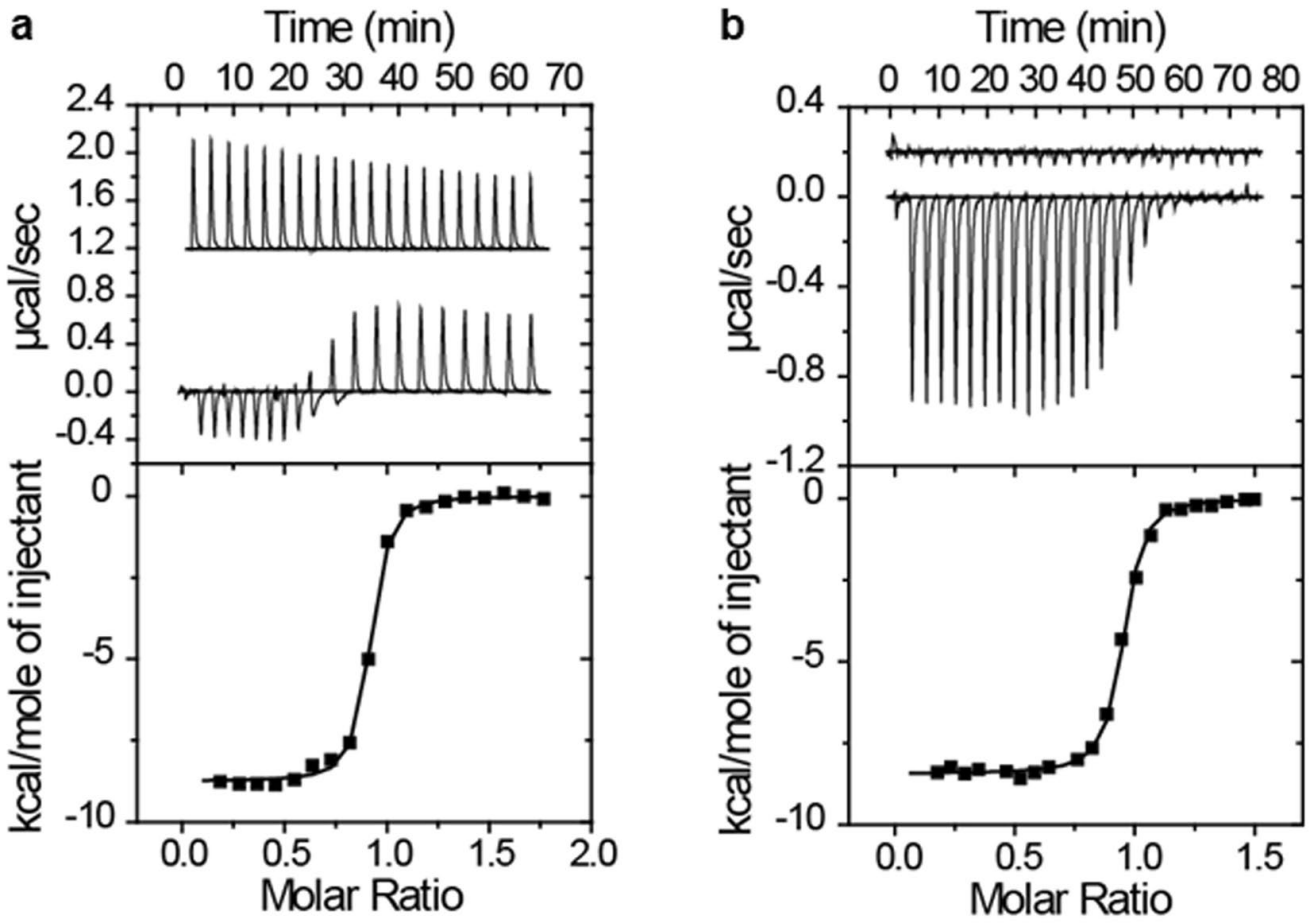
Isothermal calorimetry titrations for the binding of OsGID1/SLR1(M28-A112). Top panels: (a) Twenty 10 μl injections of 400 μM SLR1(M28-A112) into a buffer solution (upper titration curve) or a buffer solution containing 50 μM OsGID1/GA_3_ (lower titration curve). (**b**) Twenty 10-μl injections of 400 μM OsGID1/GA_3_ into a buffer solution (upper titration curve) or a buffer solution containing 50 μM SLR1 (lower titration curve). The titration curves of the upper panels are plotted as the power supplied to or removed from the sample cell that is needed to maintain a constant temperature at 30 °C versus time. The buffer was 20 mM sodium phosphate (pH 7.5), 150 mM NaCl, 2.0 mM β-ME, and 0.1 mM GA_3_. The titration curves of the bottom panels have been integrated and normalized. The heats of reaction for the buffer blanks were first subtracted from the experimental data. The solid lines are the best nonlinear least-squares fit of the data.

An ITC experiment provides a complete thermodynamic description—including the stoichiometry and the values of free energy (Δ*G*), enthalpy (Δ*H*), and entropy (Δ*S*) changes—of a binding reaction^17^. Correlating such thermodynamic data with a structural description increases our understanding of the molecular recognition process involved in complex formation and maintenance. Therefore, we used ITC to determine the stoichiometries and thermodynamic binding constants of OsGID1/GA_3_ and truncated SLR1 mutants. The SLR1 variants used were SLR1(M1-H327), SLR1(M1-A172), SLR1(M28-A112), SLR1(M1-E104) and three additional constructs, SLR1(M28-A112)ΔDELLA(E40-D56), SLR1(M28-A112) ΔTVHYNP(L93-E104), and SLR1(M28-A112) ΔSPACE(G69-F83), which are abbreviated SLR1(M28-A112)ΔDELLA, SLR1(M28-A112) Δ(TVHYNP), and SLR1(M28-A112) ΔSPACE, respectively, throughout the remainder of the paper. An exploratory control experiment—the serial addition of 10 μl aliquants of 0.4 mM SLR1(M28-A112) into 1.4482 ml of buffered solution that lacked an OsGID1/GA_3_ complex and was contained in the calorimeter cell thermostated at 30 °C—proved to be greatly endothermic (Fig. 3a). Heat absorption accompanies the dissociation of protein complexes. The large positive heat of dilution observed when a small volume of a solution containing SLR1(M28-A112) was injected into the calorimetry cell is evidence for the dissociation of an oligomer. For such a dilution series, with successive injections, less heat is absorbed because as the protein concentration increases, the equilibrium shifts to the oligomeric state. After integration and normalization, the thermal dilution profile was analysed using a nonlinear regression technique^18^ to give estimates of the dimerization constant (*K*_dim_ = 1.6 mM) and enthalpy of dimerization (Δ*H*_dim_ = −66 kcal/mol). To avoid the complication caused by dissociation of the SLR1(M28-A112) dimer, we instead titrated 10 μl aliquants of a 0.4 mM OsGID1/GA_3_ solution into the calorimeter cell containing 1.4482 ml of a solution containing 50 μM SLR1(M28-A112). Using this configuration, the titration did not generate a large positive heat of dilution (Fig. 3b). The thermodynamic constants from these two experiments are summarized in Table 1. To avoid introducing a large endothermic heat of dilution term into the heat measurements, all other titrations were performed by adding OsGID1/GA_3_ to the SLR1 variant contained in the calorimeter cell.

**Table 1:** Thermodynamic parameters and binding constants for OsGID1/GA_3_/SLR1 complex formation derived from the ITC experiments of Fig. 3.

Syringe	Sample cell	*K*_b_ M^−1^	*n*	Δ*H* kcal/mole	−*T*Δ*S* kcal/mole	Δ*G* kcal/mole
SLR1	GID1	1.2 × 10^7^	0.88	−8.75	−1.07	−9.82
GID1	SLR1	1.1 × 10^7^	0.92	−8.44	−1.33	−9.77

Next, to determine which bioactive GA was most suitable for the ITC experiments, we assayed the effects of various bioactive GAs on the OsGID1 and SLR1(M28-A112) interaction by titrating OsGID1 solutions containing GA_4_, GA_7_, GA_1_, or GA_3_ into solutions of SLR1(M28-A112). The resulting ITC profiles are indistinguishable (Fig. S4), suggesting that the interaction of an OsGID1/GA complex and SLR1(M28-A112) is independent of the chemical nature of the bioactive GA that is present. Parenthetically, the affinity of OsGID1 for a GA is dependent on the GA: for GA_4_, *K*_b_ = 5.2 × 10^6^ M^−1^; for GA_7_, *K*_b_ = 4.2 × 10^6^ M^−1^; for GA_1_, *K*_b_ = 5.2 × 10^5^ M^−1^; and for GA_3_, *K*_b_ = 2.8 × 10^5^ M^−1^. Since the chemical nature of the bioactive GA is not important, we continued to use GA_3_ in all additional titrations.

**Figure 4:**
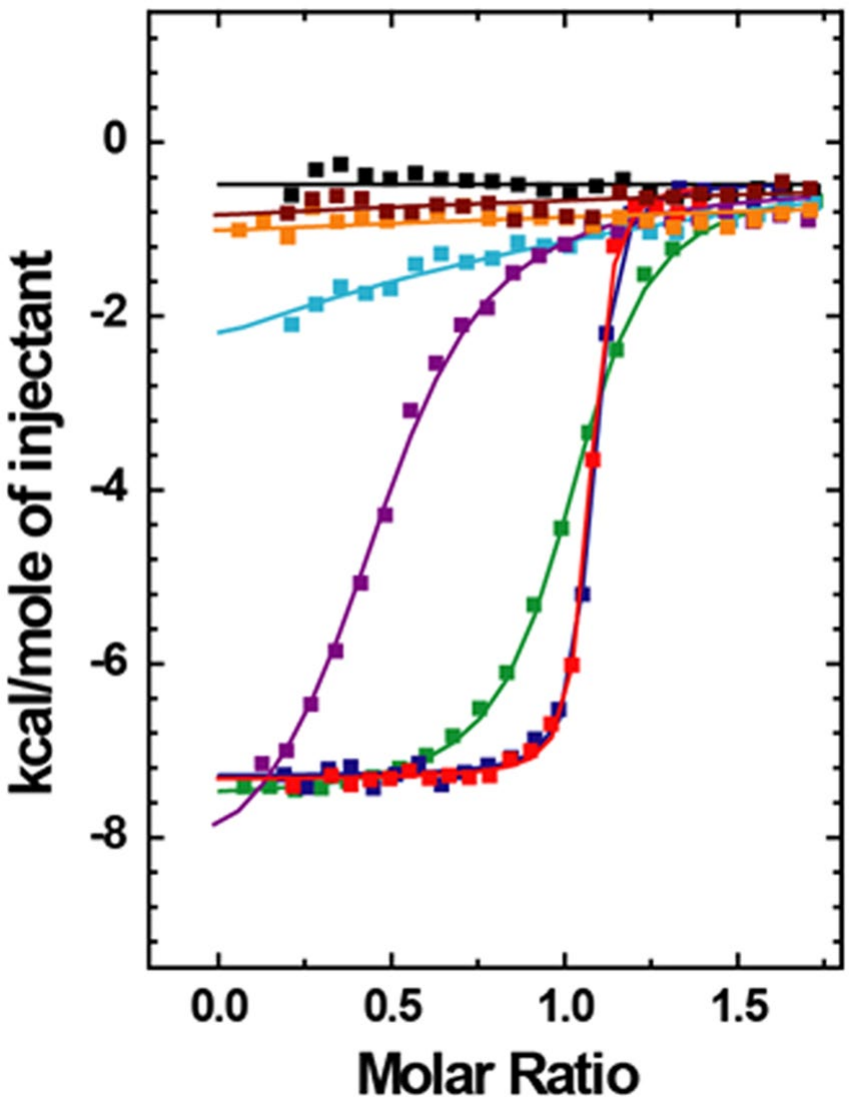
Isothermal calorimetry titrations for the binding of OsGID1/GA_3_ and various SLR1 mutants. Integrated and normalized titration curves for OsGID1/GA_3_ and SLR1(M1-H327) (green); SLR1(M1-A172) (blue); SLR1(M28-A112) (red); SLR1(M28-A112)ΔSPACE (purple); SLR1(M28-A112) ΔDELLA (brown); SLR1(M28-A112) ΔTVHYNP (orange); SLR1(M1-E104) (cyan); and SLR1(M28-A112) without GA_3_ (black). All titrations were performed at 30 °C using 50 μM SLR1 mutants and 400 μM OsGID1/GA_3_ in 20 mM sodium phosphate (pH 7.5), 150 mM NaCl, and 2.0 mM β-ME.

The integrated and normalized plots of the heat released versus the molar ratio of OsGID1/GA_3_ to SLR1 truncation mutants are shown in Fig. 4. The derived values of *K*_b_, Δ*H*, *T*Δ*S*, and Δ*G* are listed in Table 2. The mutants SLR1(M1-H327), SLR1(M1-A172), and SLR1(M28-A112) bind OsGID1/GA_3_ tightly, and the binding of these three mutants is enthalpically driven. As the three Δ*H* values are very similar, the noncovalent interactions between OsGID1/GA_3_ and each of the three mutants are likely to be very similar. Although on the basis of the Δ*H* values, it appears that these SLR1 variants bind OsGID1/GA_3_ similarly, the presence of at least some of the SLR1 residues between residues 173 and 327 causes an order of magnitude decrease in the binding constant. It is therefore possible that residues within the A173-H327 sequence inhibit binding to OsGID1 *in vitro*. Within experimental error, SLR1(M28-A112) and SLR1(M1-A172) bind OsGID1/GA_3_ with the same affinity, which suggests that the sequence M1-V27 is not needed for GID1 binding. The ITC results also show that SLR1(M28-A112) is the shortest sequence that can bind OsGID1/GA_3_ with high affinity. Conversely, SLR1(M28-A112) ΔSPACE has a lower affinity for OsGID1/GA_3_; SLR1(M28-A112) ΔDELLA and SLR1(M28-A112) ΔTVHYNP apparently do not bind OsGID1/GA_3_ at all (Fig. 4, Table 2). The absence of the S105–A112 sequence decreases the binding constant dramatically, as noted previously, to only 0.04% of that found for SLR1(M28-A112). These results suggest that the DELLA and TVHYNP sequences and residues S105-A112 are required for tight binding.

**Table 2:** The thermodynamic and binding constants derived from ITC experiments for OsGID1/GA_3_/SLR1 variants.

SLR1	*K*_b_ M^−1^	*n*	Δ*H* kcal/mole	−*T*Δ*S* kcal/mole	Δ*G* kcal/mole
SLR(M1-H327)	2.0 × 10^6^	1.01	−7.1	−1.7	−8.8
SLR(M1-A172)	2.8 × 10^7^	1.06	−6.8	−3.5	−10.3
SLR(M28-A112)	2.9 × 10^7^	1.06	−6.9	−3.5	−10.4
SLR(M28-A112)**Δ**SPACE	3.9 × 10^5^	0.50	−8.3	0.6	−7.8
SLR(M28-A112)**Δ**DELLA	n.d.	n.d.	n.d.	n.d.	n.d.
SLR(M28-A112)** Δ**YVHYNP	n.d.	n.d.	n.d.	n.d.	n.d.
SLR1(M1-E104)	1.1 × 10^4^	1.0	−8.4	2.7	−5.6

n.d., not determined.

The derived *n* values for all the ternary complexes are approximately 1, with the exception of the complex involving SLR1(M28-A112)ΔSPACE, which has an *n* value of 0.5, suggesting that two molecules of SLR1(M28-A112)ΔSPACE bind to one of OsGID1/GA_3_. As the deletion of residues G69-F83 (SPACE) decreases the separation of the DELLA and TVHYNP residues, it appears that within one molecule of SLR1(M28-A112)ΔSPACE, the two motifs no longer can explore the space needed to dock simultaneously to OsGID1/GA_3_.

### ^15^N and ^13^C NMR spectroscopy probing the conformations of SLR1(M28-A112) in the free and OsGID1/GA_3_-bound states

**Figure 5:**
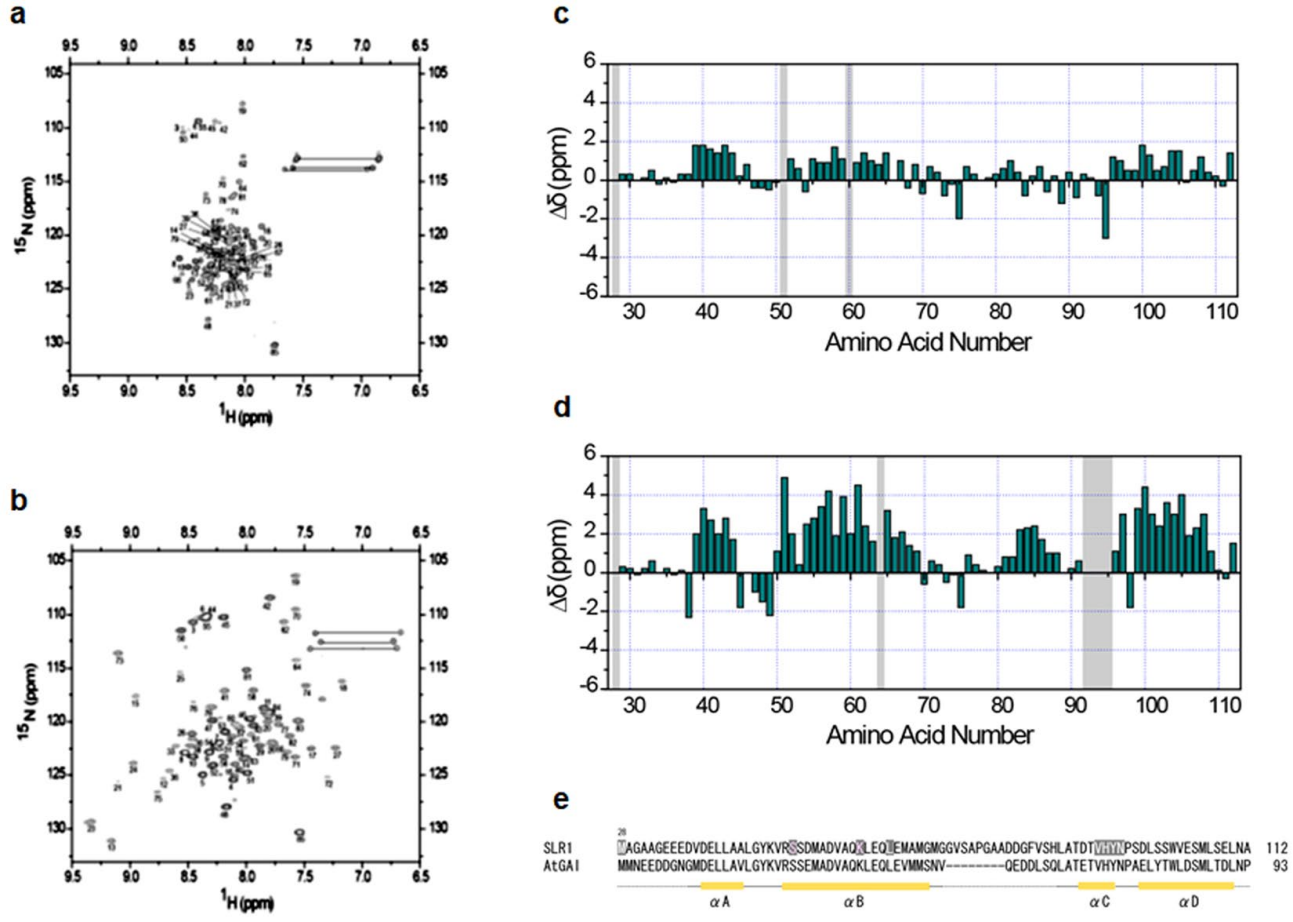
NMR spectroscopy of SLR1. Two-dimensional ^1^H-^15^N HSQC spectra of ^15^N-labelled SLR1(M28-A112) (**a**) in the absence and (**b**) presence of unlabelled OsGID1/GA_3_. Residue assignments for the backbone amide resonances are shown. Horizontal lines connect the side chain amide signals of the Asn and Gln residues. ^13^Cα chemical shift difference versus residue number for SLR1(M28-A112) (**c**) in the absence and (**d**) presence of unlabelled OsGID1/GA_3_. (**e**) Partial sequence alignment of SLR1(M28-A112) and GAI with the secondary structure that predicted the chemical shift index (see main text).

NMR spectroscopy is a powerful tool for the study of the structure and dynamics of a protein or protein complex in solution^19,20^. This technique is particularly powerful when applied to dynamic or flexible systems, such as partially folded, molten globular proteins, which are not amenable to X-ray crystallography. To further clarify the conformational states of SLR1(M28-A112) when free and when bound to OsGID1/GA_3_, we acquired the NMR spectra of these species. The ^1^H-^15^N HSQC NMR spectrum of free SLR1(M28-A112) is very similar to that expected for a disordered protein (Fig. 5a). Conversely, the cross peaks of the ^1^H-^15^N HSQC NMR spectrum of SLR1(M28-A112) acquired when unlabelled OsGID1/GA_3_ is present are well dispersed (Fig. 5b). Therefore, when bound to OsGID1/GA_3_, SLR1(M28-A112) acquires and maintains a defined tertiary structure that is absent in the free state. Murase *et al*. also found that the structure of GAI(Q11-Q113) is well defined and contains four α-helices when it is part of the ternary complex, whereas it is disordered when free in solution^13^. To determine what, if any, secondary structure is present in the bound and free forms of SLR1(M28-A112), the deviations of the ^13^Cα chemical shifts from typical random coil values were determined^21^. Figure 5c and d are plots of the Cα chemical shift deviations for free and bound SLR1(M28-A112), respectively. Although the appearance of the ^1^H-^15^N-HSQC spectrum of the free SLR1(M28-A112) is very similar to that expected for a disordered protein, the ^13^Cα resonances of Glu39-Ala44 and Ala55-Glu62 are shifted downfield somewhat (ca. 2.0 ppm), suggesting that the protein is not completely disordered. The Cα resonances of α-helices are typically shifted downfield by ca. 3.1 ppm compared with those of a disordered structure^21^. Therefore, these SLR1 residues may have a propensity to form helices even in the absence of other stabilizing influences when SLR1(M28-A112) is free in solution. However, the signature α-helical NOEs between HNi and Hαi-3 resonances were not observed. On the other hand, large positive deviations (ca. 3.0–4.0 ppm) in the ^13^Cα chemical shifts of the residues of E39-A44, R50-G69, D81-A88, and L99-E109 are observed when SLR1(M28-A112) and OsGID1/GA_3_ are both present, suggesting that these sequences form α-helices. Except for the SLR1 D81–A88 sequence, these SLR1 sequences correspond to GAI sequences that are helical when part of the *Arabidopsis* ternary complexes. The signals belonging to residues V92-N95 contained within the TVHYNP sequence are absent from the ^1^H-^15^N-HSQC spectrum, suggesting that they are broadened beyond observation and that the fluctuation rates of the residues are comparable to the NMR time scale. Taken together, the NMR results indicate that SLR1(M28-A112) is a natively unfolded structure with some, albeit not very stable, secondary structure that acquires a well-defined, three-dimensional fold when bound to OsGID1/GA_3_.

## Discussion

The three-dimensional structures of two *Arabidopsis* GID1/GA/DELLA protein complexes have been reported; however, to date, the structure of GID1/GA/DELLA from rice or other plants has not been solved. Furthermore, the structure of a free DELLA protein or a free GID1 has not been described in detail, so we understand little about how the two proteins recognize each other. Consequently, for this article, primarily using SLR1 truncation mutants, we partially characterized SLR1 structurally when free in solution and when bound to OsGID1/GA_3_; additionally, we characterized the thermodynamics of the binding of OsGID1/GA_3_ to various SLR1 mutants. To do so, we analysed data obtained from gel filtration chromatography, SAXS experiments, ITC, and NMR spectroscopy.

### Structural characterization of SLR1 in the free state

The following results demonstrate that SLR1 tends to self-aggregate. (i) All the Trx-SLR1 variants elute from a Superdex 200 column at positions that correspond to MWs much larger than expected theoretically. For example, Trx-SLR1(M28-A112) behaved chromatographically as a dimer (Fig. 1). (ii) As shown by ITC, when a sample of SLR1(M28-A112) is diluted into a buffer blank, heat is absorbed. The integrated calorimetric titration curve is consistent with a dimer-to-monomer equilibrium that is shifted to the monomeric state upon dilution. (iii) Using data obtained from the SAXS experiment, the MW of SLR1(M28-A112) is estimated to be 12.9 kDa, which is approximately 1.5-fold larger than the theoretical MW (8.9 kDa) (Fig. S3). From these results, the oligomerization state of SLR1 might be depend on the solution conditions, such as concentration and temperature.

Although interpretation of the ^1^H-^15^N-HSQC spectrum (Fig. 5a) and the Kratky plot (Fig. S3B) of SLR1(M28-A112) suggests a disordered protein, the downfield shifts of the ^13^Cα resonances (ca. 2.0 ppm; Fig. 5c) of residues E39-A44 and A55-E62 are indicative of marginally stable helices.

### A conformation change occurs when SLR1(M28-A112) binds OsGID1/GA_3_

The following results demonstrate that SLR1(M28-A12) and GID1/GA_3_ associate with a stoichiometry of one to one. (i) We have shown, using ITC, that SLR1(M28-A112) binds with high affinity and a stoichiometry of one-to-one to OsGID1/GA_3_ (Fig. 4). ii) When Trx-SLR1(M28-A112) is incubated with OsGID1/GA_3_, a single protein peak is found that elutes from a Superdex 200 column at a position that corresponds to the expected MW of a Trx-OsGID1/GA_3_/Trx-SLR1(M28-A112) complex (Fig. 2). (iii) Analysis of the Kratky plot for a mixture containing OsGID1/GA_3_ and SLR1(M28-A112) strongly suggests that the associated proteins form a one-to-one complex that has a globular shape. Therefore, as was found for the AtGID1a/GA/GAI(Q11-Q113) crystal structures^13^, the rice complex contains one each of OsGID1/GA and SLR1.

Examination of the values of the SLR1 ^13^Cα chemical shifts of GID1/GA_3_/SLR1(M28-A112) shows that the ^13^Cα resonances of the residues of the sequences E39-A44, R50-G69, D81-A88, and L99-E109 shift ca. 3.0–4.0 ppm downfield compared with those of free SLR1(M28-A112). This shift is consistent the formation of α-helices (Fig. 5c). Therefore, while the structure of free SLR1(M28-A112) is mostly disordered, the protein acquires a defined fold when bound to its physiological target, GID1.

A significant body of evidence suggests that while intrinsically disordered proteins lack structure, they are nevertheless functional^22^. Intrinsically disordered proteins can be broadly classified into two major groups: those that are fully disordered (natively unfolded) and those that have extensive (>30–40 residues) regions that are disordered and embedded in an otherwise folded protein^23^. Natively unfolded proteins can be further subdivided into two groups, those with no ordered structure and those with some marginally stable secondary structure. The latter resemble molten globules. A molten globule is a stable, partially folded protein and is often found when the solution conditions are mildly denaturing^24^. Accordingly, SLR1 is a natively unfolded protein with some secondary structure resembling a molten globule. Notably, disordered proteins often bind to a ligand, such as a protein, nucleic acid, or membrane, attain a well-defined shape, and then participate in cell cycle control or in transcriptional or translational regulation processes. As we have shown, SLR1 folds upon binding to OsGID1/GA, allowing the GA signal to be transmitted farther down the GA pathway.

### Summarizing the GA signalling mechanism

Our gel filtration, SAXS, ITC and NMR experimental results support the concept that upon binding OsGID1/GA, SLR1 attains a defined fold. Free SLR1 exists as an oligomeric, natively unfolded protein that contains some unstable helical secondary structure. Although Murase *et al*. concluded that GAI(Q11-Q113) is completely unfolded on the basis of its CD spectrum, SLR1(M12-A112) appears to contain two unstable α-helices. It is possible that—given the propensity of E39-A44 (corresponding to αA in GAI) to form a helix—it may initially recognize OsGID1/GA and, as shown for the AtGID1/GA/GAI(Q11-Q113) complex, bind at the crevice that is formed by the OsGID1 N-terminal extension helix and the core OsGID1 domain (Fig. S2). Furthermore, A55-E62 may also be involved in the initial binding, as by analogy with the αB helix of the *Arabidopsis* DELLA protein, A55-E62 should interact directly with the OsGID1 extension helix. Both the corresponding GAI α-helices interact with the AtGID1a N-terminal extension helix. Therefore, as suggested by Murase *et al*., the N-terminal extension of GID1 would act as a conformational switch that when turned on, stabilizes not only the GA binding but also the subsequent binding of SLR1^13^.

## Methods

### Cloning and purification of OsGID1 and SLR1 mutants

The cloning, expression, and purification of OsGID1 and Trx-OsGID1 have been described elsewhere^11^. Starting with SLR1 cDNA and primers—with sequences conforming to the requirements of the Gateway system (Table S1)—truncated SLR1 cDNAs were prepared. For each truncated SLR1, PCR-amplified cDNA was inserted into a pENTR/SD/D-TOPO vector using pENTR/SD/D-TOPO Cloning Kit reagents (Invitrogen, Carlsbad, CA), and expression plasmids were then constructed using LR Reaction kit reagents (Invitrogen, Carlsbad, CA). Each SLR1 cDNA was cloned into a pDEST-trx expression vector^25^ to create the DNA for the corresponding thioredoxin–hexahistidine (Trx–(His)_6_-) SLR1 fusion protein.

The recombinant SLR1 truncated mutant proteins were expressed in *E. coli* upon the addition of 1.0 mM IPTG (final concentration), followed by a 16 hour incubation at 18 °C. The Trx-SLR1 truncated mutants were each chromatographed over a 5 ml HiTrap Chelating HP column (GE Healthcare Bio-Sciences, Picataway, NJ) at a flow rate of 5 ml per minute. Each eluted protein was dialyzed against 20 mM sodium phosphate (pH 7.5), 150 mM NaCl, and 2 mM β-mercaptoethanol (ME) and then chromatographed over a HiLoad 26/60 Superdex 200 pg column (GE Healthcare Bio-Sciences, Picataway, NJ) at a flow rate of 1 ml per minute.

To remove the Trx-(His)_6_ tags, proteins were incubated with thrombin (0.75 U/mg protein; Novagen, Madison, MI) and then chromatographed over a 5 ml of HiTrap Chelating HP column. Each flow-through fraction was dialyzed against 20 mM sodium phosphate (pH 7.5), 150 mM NaCl, and 2 mM β-ME and then chromatographed over a HiLoad 26/60 Superdex 75 pg column (GE Healthcare Bio-Sciences, Picataway, NJ). The purified proteins were concentrated over Amicon Ultra-15 centrifugal filters (Millipore, Bedford, MA).

### Gel filtration chromatography

For each chromatograph, purified Trx-OsGID1 and a Trx-SLR1 truncation mutant were incubated together at 4 °C for 30 min with 10^−4^ M GA_3_ in 20 mM sodium phosphate (pH 7.5), 150 mM NaCl, and 2 mM β-ME. One hundred microlitres of each mixture was chromatographed over a Superdex 200 10/300 GL column (GE Healthcare, Picataway, NJ) in the same buffer as used for its incubation. The individual proteins were chromatographed using the same conditions. Fractions of 0.5 ml were collected, and those containing protein were subjected to SDS-PAGE.

### SAXS measurements

For SAXS measurements, the CCD-based X-ray detector (Hamamatsu Photonics K. K., Hamamatsu, Japan)^26^ installed at beamline BL15A of the Photon Factory was used. The sample-to-detector distance was 1.0 m, and the X-ray wavelength (λ) was 1.5 Å. The scattering vector, *q*, was monitored between 0 Å^−1^ and 0.30 Å^−1^, where *q* = 4πsin*θ*/λ and 2*θ* is the scattering angle. For the SAXS measurements, purified OsGID1 and SLR1(M28-A112), each at a concentration of approximately 3.0 mg/ml, were individually dialyzed for approximately 16 hours against 20 mM sodium phosphate (pH 7.5), 150 mM NaCl, 2.0 mM β-ME, and 0.1 mM GA_3_. The scattering profiles of OsGID1, SLR1(M28-A112), and OsGID1/GA_3_/SLR1(M28-A112) were each measured for 24 seconds per frame at 25 °C. Two or three frames were merged to improve the signal-to-noise ratio. The data were corrected for image distortion, non-uniform sensitivity, and contrast reduction using an X-ray image intensifier^27^.

The values of the radii of gyration (*R*_g_s) and the forward scattered intensities, *I*(0)s, were obtained from the Guinier approximation: *I*(*q*) = *I*(0) exp(−*q*^2^ *R*_g_^2^/3), where *I*(*q*) is the experimentally measured scattered intensity^28^. The data points in the small *q* region, i.e., *qR*_g_ ≤ 1.3, were used for the calculation. The distance distribution function *p*(*r*) was calculated using the program Saxfft3n^29^.

### **Calorimetric studies**

Isothermal titration calorimetry (ITC) experiments were performed with a MicroCal VP-ITC calorimeter (MicroCal, Northampton, MA). The instrument’s design and operation have been described in detail elsewhere^17^. For the titrations, OsGID1/GA_3_ and the SLR1 truncation mutants were first individually dialyzed for approximately 16 hours against 20 mM sodium phosphate (pH 7.5), 150 mM NaCl, 2.0 mM β-ME, and 0.1 mM GA_3_. For a given experiment, 10 μl aliquants of a 400 μM OsGID1/GA_3_ solution were added via a 300 μl syringe to a sample cell that contained 1.4482 ml of a stirred solution equilibrated at 30 °C and contained an SLR1 variant at a concentration of 50 μM. Prior to experimentation, the instrument with sample and reference cells containing water was thermally equilibrated overnight. Stable baselines were defined as those with rms noise levels of less than 5 ncal s^−1^. The heat of dilution caused by injection of OsGID1/GA_3_ into a buffer solution that did not contain protein was measured under otherwise identical buffer, injection, and temperature conditions. The heat of dilution was subtracted from the heat change that occurred when protein was present in the sample cell. Nonlinear fitting of the data was performed using MicroCal Origin 7.0 (Origin-Lab Corporation, Northampton, MA). The parameters that were varied to minimize the standard deviation of the fit to the experimental data were the binding constant (*K*_b_), the enthalpy change (Δ*H*), and the number of binding sites per protein molecule (stoichiometry, *n*). The derived values for *K*_b_, Δ*H*, and *n* were then used to calculate the changes in free energy (Δ*G*) and entropy (Δ*S*).

### **NMR spectroscopy**

All NMR spectra were recorded at 30 °C using a Bruker AV600 spectrometer equipped with a 5 mm inverse triple-resonance cryo-probehead with three-axis gradient coils. The sample solutions were 1.0 mM ^13^C/^15^N-labelled SLR1(M28-A112), 20 mM sodium phosphate (pH 7.0), and 150 mM NaCl with or without 1.0 mM OsGID1/GA_3_. The ^1^H, ^13^C, and ^15^N sequential resonance assignments used data obtained from the following 2D double resonance and 3D double and triple resonance through-bond correlation experiments: 2D ^1^H-^15^N HSQC; 3D ^15^N-separated HOHAHA-HSQC; 3D HNHA; 3D HNCO; 3D CBCA(CO)NH; and 3D HNCACB^30^. All spectra were processed using NMRPipe software^31^ and were analysed using Sparky software^32^. ^1^H, ^13^C, and ^15^N chemical shifts were referenced directly to HDO (4.64 ppm at 30 °C), indirectly to TSP (^13^C)^33^, and to liquid ammonia (^15^N), respectively^34^.

## Electronic supplementary material

Dataset 1

## References

[1] Olszewski N, Sun TP, Gubler F (2002). Gibberellin signaling: biosynthesis, catabolism, and response pathways. Plant Cell.

[2] Richards DE, King KE, Ait-Ali T, Harberd NP (2001). How Gibberellin Regulates Plant Growth and Development: A Molecular Genetic Analysis of Gibberellin Signaling. Annu Rev Plant Physiol Plant Mol Biol.

[3] MacMillan J (1997). Gibberellin biosynthesis from gibberellin A12-aldehyde in endosperm and embryos of Marah macrocarpus. Plant Physiol.

[4] Ueguchi-Tanaka M, Nakajima M, Motoyuki A, Matsuoka M (2007). Gibberellin receptor and its role in gibberellin signaling in plants. Annu Rev Plant Biol.

[5] Schwechheimer C (2008). Understanding gibberellic acid signaling–are we there yet?. Curr Opin Plant Biol.

[6] Hirano K, Ueguchi-Tanaka M, Matsuoka M (2008). GID1-mediated gibberellin signaling in plants. Trends Plant Sci.

[7] Ueguchi-Tanaka M (2005). Gibberellin Insensitive Dwarf1 encodes a soluble receptor for gibberellin. Nature.

[8] Itoh H, Ueguchi-Tanaka M, Sato Y, Ashikari M, Matsuoka M (2002). The gibberellin signaling pathway is regulated by the appearance and disappearance of SLENDER RICE1 in nuclei. Plant Cell.

[9] Sasaki A (2003). Accumulation of phosphorylated repressor for gibberellin signaling in an F-box mutant. Science.

[10] Ueguchi-Tanaka M, Hirano K, Hasegawa Y, Kitano H, Matsuoka M (2008). Release of the repressive activity of rice DELLA protein SLR1 by gibberellin does not require SLR1 degradation in the gid2 mutant. Plant Cell.

[11] Ueguchi-Tanaka M (2007). Molecular interactions of a soluble gibberellin receptor, GID1, with a rice DELLA protein, SLR1, and gibberellin. Plant Cell.

[12] Bolle C (2004). The role of GRAS proteins in plant signal transduction and development. Planta.

[13] Murase K, Hirano Y, Sun TP, Hakoshima T (2008). Gibberellin-induced DELLA recognition by the gibberellin receptor GID1. Nature.

[14] Kataoka M, Hagihara Y, Mihara K, Goto Y (1993). Molten globule of cytochrome c studied by small angle X-ray scattering. J Mol Biol.

[15] Semisotnov GV (1996). Protein globularization during folding. A study by synchrotron small-angle X-ray scattering. J Mol Biol.

[16] Kataoka M, Kuwajima K, Tokunaga F, Goto Y (1997). Structural characterization of the molten globule of alpha-lactalbumin by solution X-ray scattering. Protein Sci.

[17] Velazquez-Campoy, A., Ohtaka, H., Nezami, A., Muzammil, S. & Freire, E. Isothermal titration calorimetry. *Curr Protoc Cell Biol* Chapter 17, Unit17. **18**, 10.1002/0471143030.cb1708s23 (2004). 10.1002/0471143030.cb1708s23 18228446

[18] McPhail DCA (1997). Thermodynamics and kinetics of dissociation of ligand-induced dimers of vancomycin antibiotics. J. Chem.Soc.-Faraday Trans..

[19] Redfield C (2004). NMR studies of partially folded molten-globule states. Methods Mol Biol.

[20] Redfield C (2004). Using nuclear magnetic resonance spectroscopy to study molten globule states of proteins. Methods.

[21] Wishart DS, Sykes BD (1994). Chemical shifts as a tool for structure determination. Methods Enzymol.

[22] Fink AL (2005). Natively unfolded proteins. Curr Opin Struct Biol.

[23] Tompa P (2002). Intrinsically unstructured proteins. Trends Biochem Sci.

[24] Pande VS, Rokhsar DS (1998). Is the molten globule a third phase of proteins?. Proc Natl Acad Sci USA.

[25] Tsunoda Y (2005). Improving expression and solubility of rice proteins produced as fusion proteins in Escherichia coli. Protein Expr Purif.

[26] Amemiya Y (1995). Large-aperture TV detector with a beryllium-windowed image intensifier for X-ray diffraction. Rev. Instrum. Methods.

[27] Ito K (2001). Photon Factory Activity Rep.

[28] Guinier, A. & Fournet, G. *Small-angle scatttering of X-rays*. *Wiley, New York* (1995).

[29] Igarashi Y (1995). Solution X-ray scattering study on the chaperonin GroEL from Escherichia coli. Biophys Chem.

[30] Clore GM, Gronenborn AM (1991). Two-, three-, and four-dimensional NMR methods for obtaining larger and more precise three-dimensional structures of proteins in solution. Annu Rev Biophys Biophys Chem.

[31] Delaglio F (1995). NMRPipe: a multidimensional spectral processing system based on UNIX pipes. J Biomol NMR.

[32] Goddard, T. D. & Kneller, D. G. SPARKY 3 University of Calfornia, San Francisco, CA (2008).

[33] Live DH, Davis DG, Agosta WC, Cowburn D (1984). J. Am. Chem. Soc..

[34] Wuthrich K, Billeter M, Braun W (1983). Pseudo-structures for the 20 common amino acids for use in studies of protein conformations by measurements of intramolecular proton-proton distance constraints with nuclear magnetic resonance. J Mol Biol.

