# Supplementary material for: Physical and thermodynamic characterization of the rice gibberellin receptor/gibberellin/DELLA protein complex
Source: Sci Rep. 2018 Dec 7;8:17719. doi: 10.1038/s41598-018-35765-x (PMC6286387; doi:10.1038/s41598-018-35765-x)
Supplement: Supplementary file 1 — Dataset 1 [file 41598_2018_35765_MOESM1_ESM.docx]

Supporting Online Material for

**Physical and thermodynamic characterization of the rice gibberellin receptor/gibberellin/DELLA protein complex**

Hongyu Xiang^a,c^, Hideyasu Okamura^a,d^, Yuichiro Kezuka^b^ and Etsuko Katoh^a,*^

^a^ Advanced Analysis Center, National Agriculture and Food Research Organization, Tsukuba, Ibaraki 305-8602, Japan

^b^ School of Pharmacy, Iwate Medical University, Yahaba, Iwate, 028-3694, Japan

^c^ Present address: School of Life Sciences, Jilin University, Qianjin Street, Changchun, 130012, China

^d^ Present address: RIKEN Center for Biosystem Dynamics Research Laboratory for Cellular Structural Biology, Tsurumi-ku, Kanagawa, 230-0045, Japan

**This PDF file includes:**

Figs. S1 to S4

Table S1


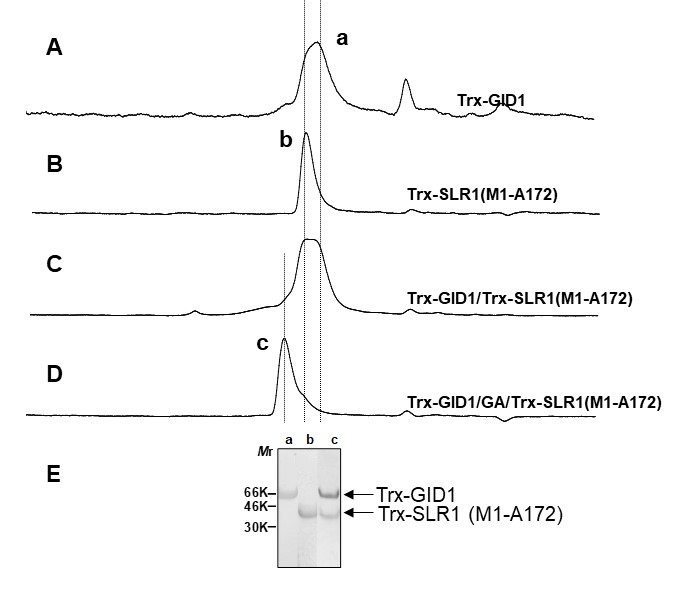


Fig. S1. *In vitro* GA-dependent Interaction of Trx-GID1 with Trx-SLR1. (A-D), Superdex-200 gel filtration profiles of Trx-GID1 (A), Trx-SLR1 (M1-A172) (B), mixture of Trx-GID1 and Trx-SLR1 (M1-A172) without GA_3_ (C), and the mixture of Trx-GID1 and Trx-SLR1 (M1-A172) with GA_3_ (D). (E); SDS-gel electrophoresis of peak a, b, c in Fig. S1 A, B, and D, respectively.


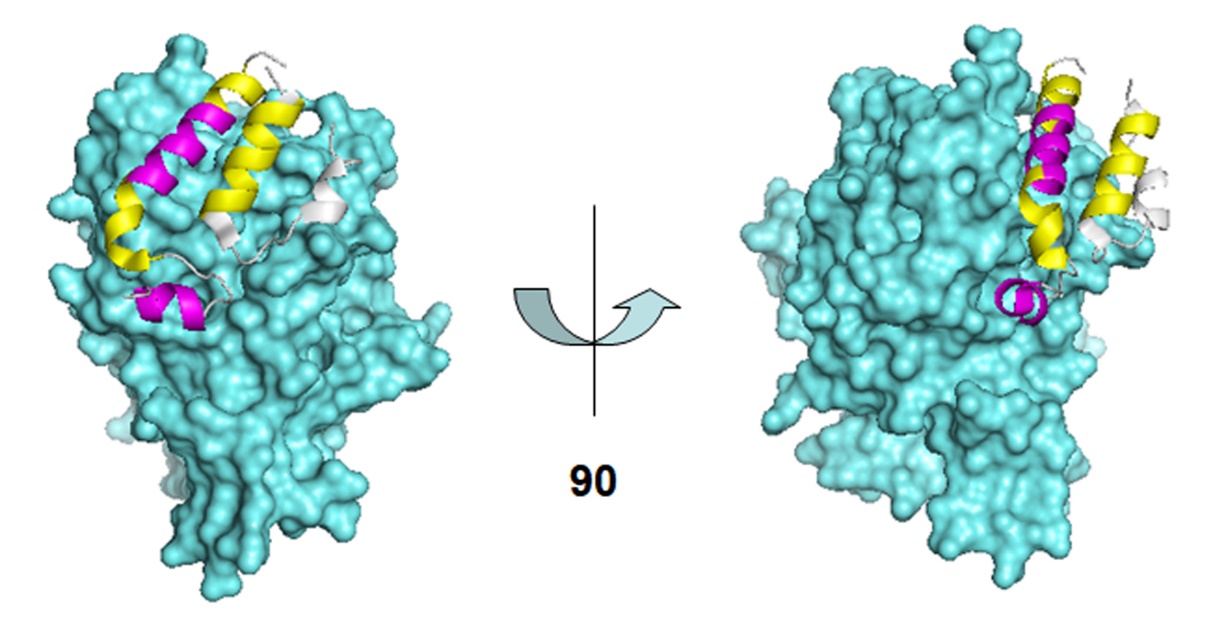


Fig. S2. Structure of the AtGID1a/GA_4_/GAI(Q11-Q113) complex^13^. The GAI backbone, depicted as a ribbon diagram, is shown bound to the AtGID1a surface (blue). Residues for which the associated chemical shift differences in Fig. 5C were found to be larger than 1.5 ppm are coloured magenta. Residues for which the associated chemical shift differences were less than 1.5 ppm (Fig. 5C) but greater than 3.0 ppm (Fig. 5D) are coloured yellow.


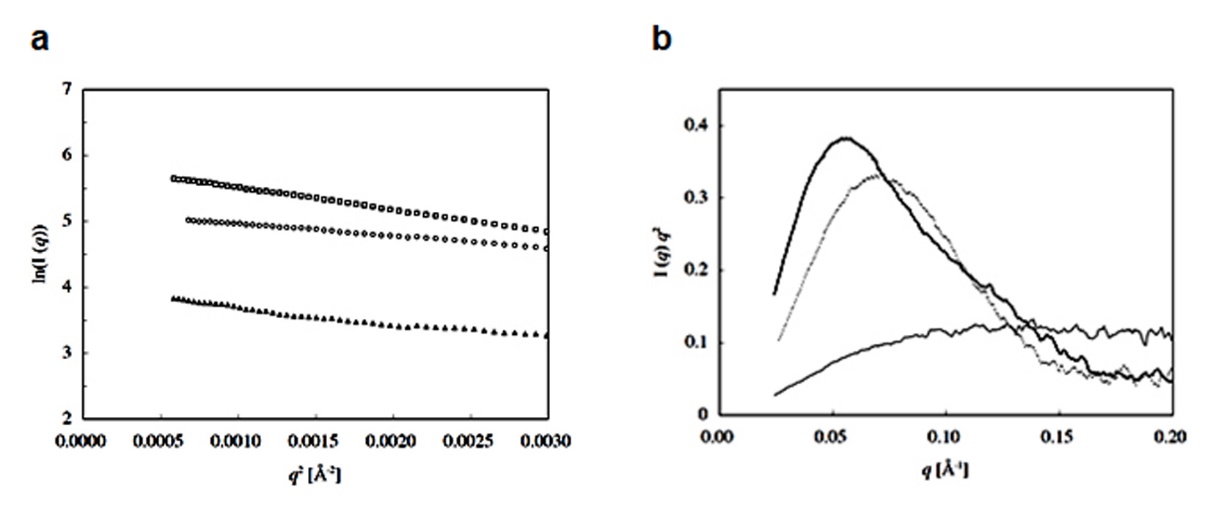


Fig. S3. Small-angle X-ray profiles. (a) Guinier plots of OsGID1/GA_3_ (squares), SLR1(M28-A112) (triangles), and OsGID1/GA_3_/SLR1(M28-A112) (circles) and (b) Kratky plots of OsGID1/GA_3_ (bold line), SLR1(M28-A112) (thin line), and OsGID1/GA_3_/SLR1(M28-A112) (dotted line). The solution pH was 7.5, and the temperature was 25 °C for all experiments.


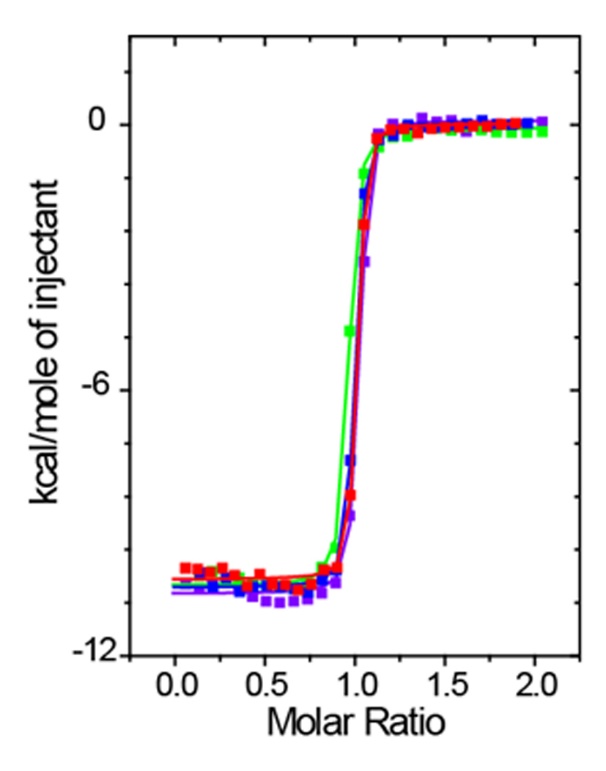


Fig. S4. Isothermal calorimetry titrations for the binding of OsGID1 and SLR1(M28-A112) in the presence of different GAs. Integrated and normalized titration curves for OsGID1, SLR1(M28-A112), GA_4_ (red), GA_7_ (violet), GA_1_ (green) or GA_3_ (blue). All titrations were performed at 30 °C using 50 μM SLR1(M28-A112) and 400 μM OsGID1 in 20 mM sodium phosphate (pH 7.5), 150 mM NaCl, 2.0 mM β-ME, and 1 mM GA.

Table S1. Primer sequences

| Primer name | Nucleotide sequence |
| --- | --- |
| SLR1(M1) F  SLR1(M28) F  SLR1(E104) R  SLR1(L110) R  SLR1(A112) R  SLR1(A172) R  SLR1(H327) R | 5’- caccATCGAAGGTCGTatgaagcgcgagtaccaagaagc - 3’  5’ - caccATCGAAGGTCGTatggcgggggcggcggg - 3’  5’ - TCACTAttactcgacccaggaggagaggtc - 3’  5’ - TCACTAgagctcggaaagcatgctctcgaccc - 3’  5’ - TCACTAcgcgttgagctcggaaagcatgctctcga - 3’  5’ - TCACTAagccgacgggtcagccgtcgccacc - 3’  5’ - TCACTAttagtgggcgaacttgaggtagggg - 3’ |

Protease sites are underlined, and the abbreviations **of** nucleotides involved in stop codons are capitalized.
